# Olfactory neurogenesis and its role in fear memory modulation

**DOI:** 10.3389/fnbeh.2023.1278324

**Published:** 2023-09-28

**Authors:** Monserrat Silvas-Baltazar, Grecia López-Oropeza, Pilar Durán, Alonso Martínez-Canabal

**Affiliations:** ^1^Licenciatura en Neurociencias, Facultad de Medicina, Universidad Nacional Autónoma de México, Ciudad de México, Mexico; ^2^Departamento de Biología Celular, Facultad de Ciencias, Universidad Nacional Autónoma de México, Ciudad de México, Mexico; ^3^Posgrado en Ciencias Biológicas, Facultad de Ciencias, Universidad Nacional Autónoma de México, Ciudad de México, Mexico

**Keywords:** neurogenesis, olfactory bulb, odor-evoked memory, piriform cortex, Proust effect, fear memory

## Abstract

Olfaction is a critical sense that allows animals to navigate and understand their environment. In mammals, the critical brain structure to receive and process olfactory information is the olfactory bulb, a structure characterized by a laminated pattern with different types of neurons, some of which project to distant telencephalic structures, like the piriform cortex, the amygdala, and the hippocampal formation. Therefore, the olfactory bulb is the first structure of a complex cognitive network that relates olfaction to different types of memory, including episodic memories. The olfactory bulb continuously adds inhibitory newborn neurons throughout life; these cells locate both in the granule and glomerular layers and integrate into the olfactory circuits, inhibiting projection neurons. However, the roles of these cells modulating olfactory memories are unclear, particularly their role in fear memories. We consider that olfactory neurogenesis might modulate olfactory fear memories by a plastic process occurring in the olfactory bulb.

## Introduction

The sense of smell is critical to generating chemical representations of the environment, as animals can detect and store odors as memory representations (Lledo et al., 2006; Stevenson and Attuquayefio, 2013). The olfactory bulb (OB) is the first brain instance of olfactory processing, while the piriform cortex (PC) enables the permanent storage of olfactory memories, which was proven by optogenetic tagging and evocation of odor memories (Meissner-Bernard et al., 2019). The roles of the OB in odor processing include acquisition, processing, and discrimination of odorant stimuli. Interestingly, the OB is one of the two brain sites where newborn neurons integrate into functional circuits throughout life (Brann and Firestein, 2014). In the OB, newborn neurons substitute mature depleted cells; therefore, this structure has a constant cellular turnover (Lledo and Valley, 2016).

The mechanism by which recently generated neurons contribute to the establishment and upkeep of olfactory memories remains poorly understood. However, they drive continuous circuitry changes in the OB, likely modulating olfactory memory expression. Odor memories are intensely evocative of other associated memories (Chu, 2000); thus, odors can function as conditioned stimuli paired with classical unconditioned stimuli (Otto et al., 1997). Previous studies showed that adult neurogenesis modulates hippocampus-dependent contextual-evoked fear memories (Seo et al., 2015; Martínez-Canabal et al., 2019; Besnard and Sahay, 2021). Therefore, a significant knowledge gap is whether the constant olfactory neurogenesis modulates fear-associated olfactory memories by altering the odor memory influence over areas like the amygdala or the hippocampal formation, critical for memory evoked fear expression.

## Olfactory bulb connectivity

Olfaction is fundamental in guiding a range of essential behaviors, from feeding to mating, directed by the chemical environment (Shipley and Ennis, 1996). Olfactory receptors detect odorant molecules on the surface of olfactory sensory neurons (OSNs) at the olfactory epithelium of the nasal mucosa. The axons of the OSNs project to the OB, a structure functioning as a primary information processing center. Golgi (1875) described the five layers that form its laminar structure.

**Figure 1:**
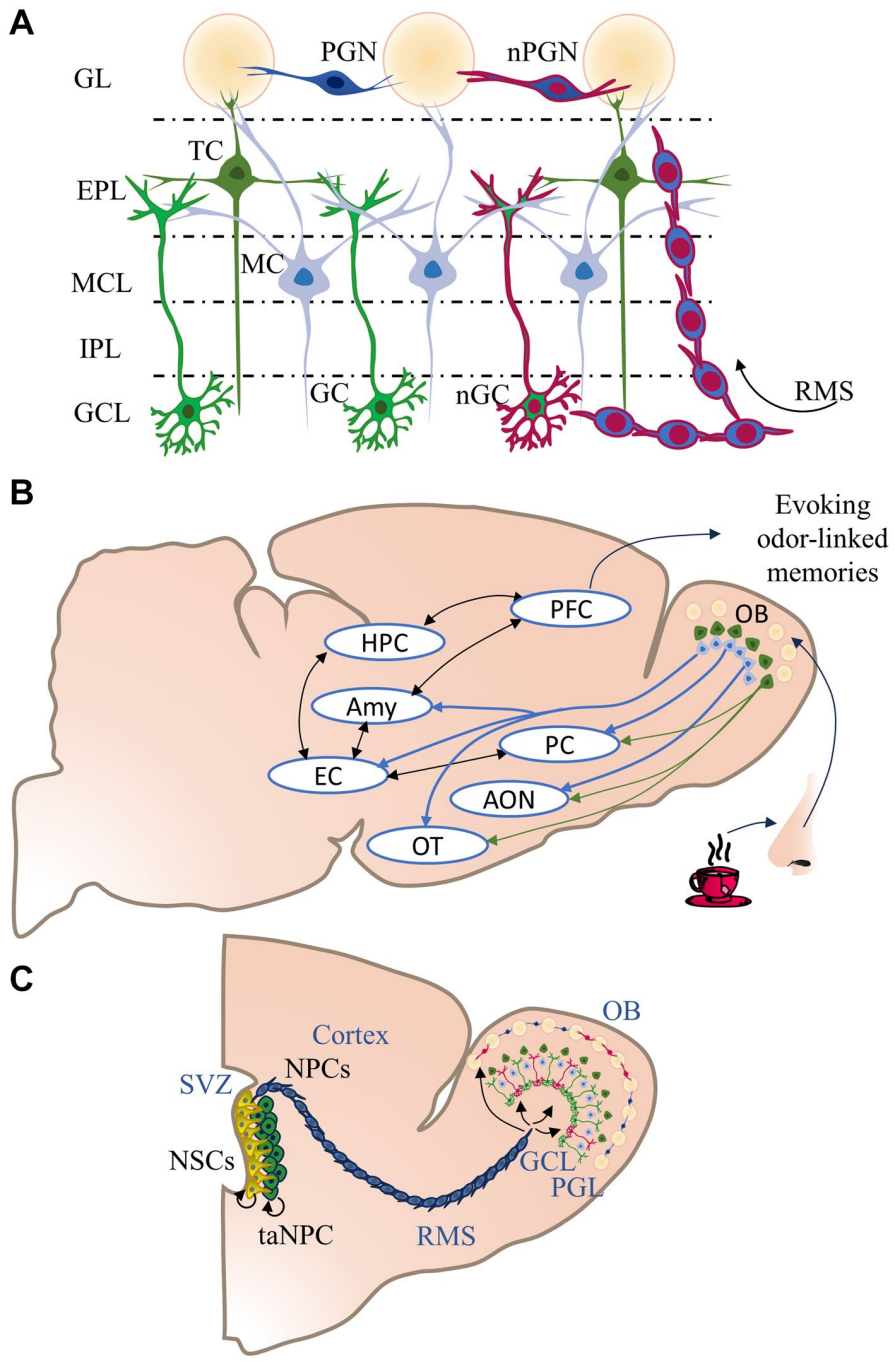
Organization, connectivity, and post-natal neurogenesis in the olfactory bulb. **(A)** The olfactory bulb (OB) has five layers: The glomerular layer (GL), where glomeruli, periglomerular cells (PGN), and newborn periglomerular cells (nPGN) are confined. The external plexiform layer (EPL) is formed by the soma of tufted cells (TC) (dark green cells), which form reciprocal connections with granule cells dendrites. The mitral cell layer (MCL) possesses the soma of mitral cells (MC) (light blue cells), which form reciprocal connections with granule cells (GC) and newborn granule cells (nGC) dendrites. MCs/TCs have axon collaterals within the internal plexiform layer (IPL). **(B)** The OB receives odorant information coming from the olfactive epithelium, then the information is processed by the internal circuity of the OB. The MCs/TCs send their outputs to the piriform cortex (PC), anterior olfactory nucleus (AON), and olfactory tubercle (OT). TC projections are specific and scarcer, while projections of MC are extensive to several areas. The MC also connects to the entorhinal cortex (EC) and the amygdala (Amy), which stores information regarding emotions such as fear and aversion. The EC communicates with the hippocampus (HPC), which encodes and processes episodic-like memories. HPC and Amy send their axons to the prefrontal cortex (PFC), which process and store Odor-episodic memories. **(C)** The OB undergoes a constant turnover of GC and PGC. The process of neurogenesis starts in the walls of the subventricular zone (SVZ). The neuronal stem cells (NSC) replicate several times and generate transient-amplifying neuronal progenitor cells (NPC), which in turn produce neuronal progenitor cells (NPC). The NPCs migrate to the OB through the rostral migratory stream (RMS). The newborn neurons integrate into the GCL or GL preexisting circuits and become nGCs or nPGCs.

OB neurons generate local engrams, organized horizontally between multiple layers: the granule cell layer (GCL), which contains granule neurons; the external plexiform layer (EPL), which contains tufted cells; the mitral cell layer (MCL), which contains mitral neurons, the internal plexiform layer (IPL), and the glomerular layer (GL), which contains the periglomerular neurons (PGN) and superficial short-axon cells (sSA) (Figure 1A). The circuits of the OB project axons to the primary olfactory cortex (OC) (Nagayama et al., 2014) (Figure 1B). The OC is a group of cerebral regions receiving primary connectivity from the OB, including the piriform cortex (PC), the olfactory tubercle (OT), the entorhinal cortex (EC), the amygdaloid nuclei (Amy), and anterior olfactory nucleus (AON). The mitral and tufted cells (MCs/TCs) of the OB transmit olfactory information to structures such as the AON, the PC, the OT, the EC, and amygdaloid nuclei (Shipley and Ennis, 1996) (Figure 1B). Both mitral and tufted cells receive inputs from glomeruli of the GL, which make reciprocal connections with PGN and receive olfactory information from the olfactive epithelium (Kosaka and Kosaka, 2005). The MCs/TCs extend their axons into the EPL, creating reciprocal synapsis with granule cell dendrites of the GCL. However, MCs and TCs establish differential excitatory connections to the OC; while the MCs present projections to the whole area of the OC, TCs present fewer and more specific connections to certain parts of the cortex (Nagayama et al., 2014).

## Olfactory bulb neurogenesis

Adult neurogenesis is the generation of newborn neurons during adult stages of life. This process only occurs in three areas of the mammalian brain: the subventricular zone (SVZ) of the lateral ventricles, the olfactory neuroepithelia, and the hippocampal subgranular zone (SGZ) (Brann and Firestein, 2014). The SVZ is the proliferative area that provides the OB with new interneurons (Brann and Firestein, 2014). Neural stem cells in the SVZ produce intermediate neural progenitor cells (NPCs) that give rise to migrating neuroblasts. These newborn cells migrate along the Rostral Migratory Stream (RMS) towards the OB, where they differentiate into granule or PGN cells (Whitman and Greer, 2009; Mobley, 2019) and integrate into the olfactory circuits (Lazarini and Lledo, 2011) (Figure 1C).

## Olfactory memory

**Figure 2:**
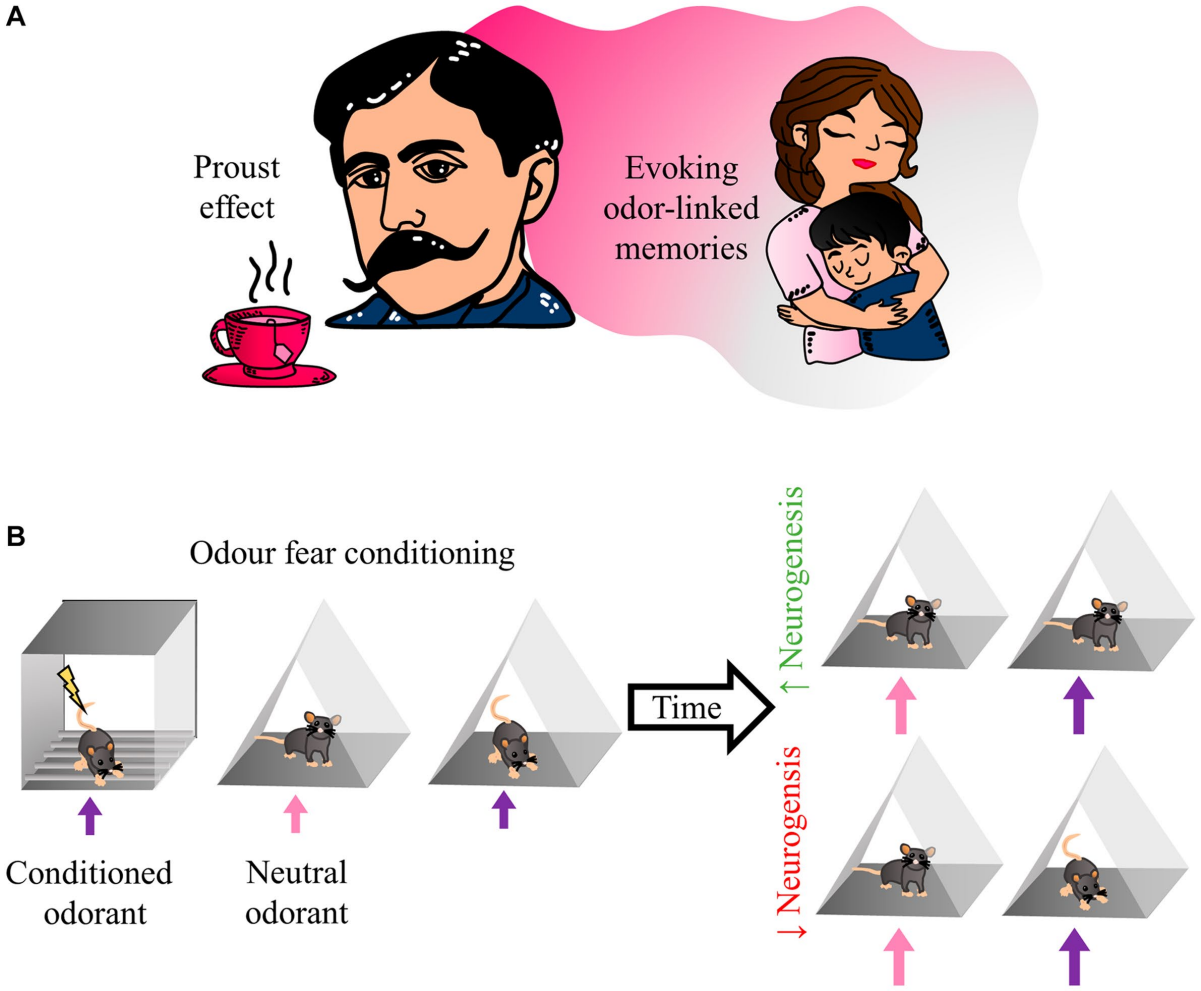
Odor fear conditioning resembles the Proust effect. **(A)** The writer Marcel Proust coined the term “involuntary memory,” referring to remembering his mother as a result of smelling a tilia tea. As a result, we name the “Proust effect,” the phenomenon in which an odorant stimulus can trigger remote episodic memories. **(B)** In the odor fear conditioning test, a conditioned odorant is paired with an aversive stimulus; the animal then expresses fear-related behavior in any place with the same odorant. However, after a month of increasing olfactory bulb neurogenesis, animals with the conditioned odorant should not show high fear responses. In contrast, animals with low neurogenesis should show normal fear responses in the presence of the conditioned odorant.

Smells provide animals with critical information about the environment, particularly about the presence of other animals, food, or threatening situations. Olfactory memories are so persistent that a single exposure can be enough to form a long-term memory, even more so when associations are present. Therefore, representations of such experiences seem significant in the behavioral responses displayed after an odor perception (Mouly and Sullivan, 2009). Olfactory memory alludes to the memory of odors and the memory associated with odors (Herz and Engen, 1996). Therefore, a perceived odor triggers the spontaneous recall of particular past events (Hackländer et al., 2018). Olfactory memories differ from other types of memory in the tendency of odors to be highly evocative of emotional memories (Roediger et al., 2017). Some authors have divided olfactory memory into two main types: odor-evoked autobiographical memory and context-dependent olfactory memory (Hackländer and Bermeitinger, 2017). In his novel “Swann’s Way” Marcel Proust evoked beautiful and painful memories of his childhood with her aunt and mother only by smelling Tilia tea with a madeleine. This effect is known as the “Proust phenomenon” and inspired the study of odor-evoked autobiographical memory (Proust, 1914; Chu, 2000) (Figure 2A). Therefore, those odors have a more significant emotional charge than other memory-evoking cues and can elicit autobiographical memory recall more efficiently than other types of cues (Herz, 1998; Koppel and Rubin, 2016).

Environmental odorants function as contextual cues in context-associated memories; therefore, retrieving olfactory information in the same context where a specific memory was encoded leads to a better performance in a memory task, as proposed by Smith (1979). Interestingly, olfactory stimuli are strong cues in human recognition memory paradigms. For instance, Cann and Ross (1989) showed images of human faces to experimental subjects and paired the faces with a pleasant, unpleasant, or no odor; 48 h later, subjects exposed to the same odorant performed better in the recognition test. Later, Schab (1990) demonstrated that environmental odors improved retrieval performance when using a specific odor in training and retrieval. Finally, animal models showed similar results. For example, Panoz-Brown et al. (2016) trained rats to remember specific odors in particular contexts and evaluated their selection of a new olfactory item that received a reward in multiple context and cue presentations. This study demonstrated that rats can remember unique events using an olfactory context association. In addition, other studies showed that environmental odorants could influence different types of memory, such as spatial memory (Schwabe and Wolf, 2009) and procedural memory (Suss et al., 2012). These multiple behavioral findings generated a growing interest in the neurobiological substrates of olfactory memory, focusing on the connectivity of the OB with other memory codification-related structures.

Neural ensembles in the PC encode olfactory memory, as this is the cortical area whose neurons receive the most OB inputs (Meissner-Bernard et al., 2019). Individual piriform neurons construct networks supporting auto-associative functions and retrieving learned information (Apicella et al., 2010; Davison and Ehlers, 2011). The PC neurons then communicate with other high-order cortical areas (Haberly, 2001), thereby supporting the construction of odor associations with episodic-like or emotional memories (Wilson and Sullivan, 2011). Other structures that receive direct OB projections are the anterior olfactory nucleus (AON), which, like the PC (Meissner-Bernard et al., 2019), participates as storage for odor engrams (Aqrabawi and Kim, 2020). The amygdala complex also receives important projections from the OB. The cortical nucleus of the amygdala participates in emotional learning and is necessary for the acquisition of conditioned fear (Sananes and Campbell, 1989; Keller et al., 2004). The medial nucleus of the amygdala is involved in olfactory memory formation (Keller et al., 2004). Also, the basolateral amygdalar complex (BLA) is involved in the learning and consolidating olfactory conditioned aversion (Kilpatrick and Cahill, 2003). The hippocampus, critical for acquiring and storing episodic-like memories, does not receive direct inputs from the OB; nevertheless, the lateral entorhinal cortex does and has direct inputs to the hippocampus (Figure 1B) (Xu et al., 2012).

## Olfactory neurogenesis roles in learning and memory

Since the discovery of olfactory neurogenesis, a primary question is what role it plays in specific olfactory functions. Nevertheless, many conflicting results exist; for instance, Lazarini et al. (2009) ablated OB neurogenesis by irradiating SVZ in mice and did not find effects in odorant discrimination and short-term olfactory memory. However, the authors detected impairment in long-term olfactory memory. Likewise, Sultan et al. (2010) studied the role of new interneurons in olfactory memory through an olfactory associative discrimination task; they described that learning influences the survival and recruitment of newborn neurons, and therefore, they infer that newborn neurons are necessary for the long-term retention of olfactory information. Finally, using a genetic ablation approach, Sakamoto et al. (2014) showed that ablation of OB neurogenesis negatively affects olfactory associative learning.

Conversely, Rochefort et al. (2002) demonstrated that odorant-enriched environments increased the number of newborn neurons in the OB, and the animals housed in these environments showed an improvement in short-term olfactory memory. Moreover, the promotion of OB neural survival with focal caspase inhibition drug delivery decreases odor discrimination (Mouret et al., 2009); furthermore, the promotion of neurogenesis with a knock-in mouse model presented an increase in olfactory memory and olfactory associative learning (Wang et al., 2015). Conversely, Bragado Alonso et al. (2019) showed that an increase in adult neurogenesis did not affect odor discrimination in basic tasks between distinct odorants; alternatively, it enhanced odor discrimination in complex tasks using similar odorants.

Although the specific functionality of adult OB neurogenesis remains unclear, a proposition posits that newborn neurons provide the OB system with effective capabilities to adapt to novel and dynamic olfactory environments (Cecchi et al., 2001; Lledo et al., 2006). Also, it seems to play a critical role in associative learning and olfactory memory (Sultan et al., 2010). However, Sultan et al. (2010) suggest that the type of task performed is critical for the output, as non-operant olfactory tasks generate a different conclusion. In these cases, neurogenesis plays a role in the maintenance and reorganization of the OB (Imayoshi et al., 2008).

## Fear learning and neurogenesis

Fear can be detected and quantified in experimental contexts, mainly through observing different reactions in animal models, such as freezing (Bouton and Bolles, 1980), the repression of operant behavior, and the startle reflex potentiation (Davis, 1990). Those behavioral expressions of fear appear after associative learning when animals learn that a conditioned stimulus (CS) predicts the onset of an aversive unconditioned stimulus (US) (Pavesi et al., 2012). Otto et al. (1997) demonstrated that an olfactory stimulus could function as an effective CS in fear conditioning in rats, following the same principles as visual or auditory stimulation paradigms. Furthermore, they showed that the amygdala plays a critical role in the acquisition and the behavioral responses to an olfactory CS (Otto et al., 2000).

The individual amygdalar nuclei play discrete roles within fear learning, e.g., odor and electric shocks activate medial (MeA) and corticomedial (CMe) nuclei (Schettino and Otto, 2001). Hence, Walker et al. (2005) further indicated that the role of the MeA was in eliciting the fear response. The lateral amygdala (LA) plays a role in the CS association; (Schettino and Otto, 2001) suggested that the olfactory information pathway for this process could be the periamygdaloid complex. The central amygdala (CeA) supports both fear learning and fear behavior output (Ciocchi et al., 2010; Keifer et al., 2015), while BLA has been related directly to olfactory fear memory consolidation, mainly through lesion approaches and its connections with the EC, and the hippocampus (Hakim et al., 2019).

The neurobiology of the olfactory fear learning formation and codification involves different OB cells, such as the activation of OSNs (Kass et al., 2013), periglomerular interneurons (Kass and McGann, 2017), and MCs/TCs (Fletcher, 2012) using calcium transients imaging. Furthermore, Ross and Fletcher (2018) used the same technology to show an increase in odor representations and activation in glomerular cells following an olfactory fear conditioning protocol.

Tonegawa et al. (2015) proposed to understand the neural circuits underlying memory encoding by distributed sets of neurons called engrams. Then, searching for the olfactory engram in the OB, Meissner-Bernard et al. (2019) used genetic cell labeling of activated neurons during olfactory fear conditioning. They reported that silencing the tagged cell assemblies caused a decrease in odor-associated escape behaviors but did not interfere with odor detection and discrimination. Alternatively, during reactivation, they observed the same diminished scanning behavior that the conditioned odor stimulus produced (Meissner-Bernard et al., 2019).

Most OB neurogenesis involves the formation of both glomerular and granule layer interneurons (Luskin, 1993); up to 94% of the newly generated neurons become GCs, the rest being both PG cells and astrocytes (Lledo and Valley, 2016). The circuits formed by these interneurons, projection cells, and cortical inputs are paramount for processing and regulating olfactory information to higher-order regions. For example, PG interneurons form the primary input and output connections in the OB through interconnections between different OSN populations (Kass and McGann, 2017) and the top-down influence of the amygdala and locus coeruleus, which could link the PG interneurons’ activity with emotional processes such as fear learning (Fast and McGann, 2017). However, the functional significance of neurogenesis in the OB as it relates to olfactory memories remains unclear. Carlén et al. (2002) mapped the activity of new neurons in response to odors using the immediate early gene c-fos. They reported that odors could activate the newly formed neurons, thus concluding that the newborn neurons integrate functionally into odor-processing circuits.

## Discussion and final remarks

The continuous addition of newborn cells to the OB during life suggests that such neurons have a critical role in maintaining the OB structure and regulating olfactory memory processing. Several studies suggest different roles of post-natal newborn neurons in OB and hippocampus in learning, discrimination, and memory consolidation. However, many details of their participation and possible roles of newborn neurons in cognition remain mysterious (Kitamura et al., 2009; Akers et al., 2014). With their interneuron phenotype, the newborn neurons regulate the activity of projecting olfactory neurons to the piriform cortex, a critical structure for engram ensemble supporting olfactory memory. The piriform cortex has strong projections to amygdaloid nuclei, which are responsible for fear memory formation and expression. The relation between the olfactory bulb, the piriform cortex, and the amygdaloid nuclei supports the notion that olfactory newborn neurons play a role in modulating fear memories conditioned to odorants. Given the inhibitory and plastic nature of the olfactory newborn neurons, they are likely to downregulate the strength of the fear memory circuits.

Bulbar neurogenesis constantly occurs within afferent networks that experience a continuous replacement of their elements. E.g. according to Watt et al. (2004), OSNs present a lifespan of ∼60 days in rodents. Hence, Lledo and Valley (2016) propose that OB neurogenesis assists in the modulation of olfactory information processing as a consequence of changes in the composition of the epithelia. In addition, the continuous presence of newborn neurons could provide fresh cells for future engrams; following this idea, OB neurogenesis would support constant adaptation in a highly dynamic environment.

Moreover, Sultan et al. (2011) hypothesized that the survival period and turnover of new neurons may compromise the stability of long-term memory. They showed that the extinction of a memory trace associating an odor and a reward promoted a substantial decrease in newborn neurons, implying that these cells participate in codifying odor-associative memories. However, those results also suggest that a natural decrease in newborn neurons associated with an odor paired with an unconditioned stimulus might cause a natural memory decay.

The OB receives inputs from the olfactory epithelium and higher-order areas; therefore, it becomes an essential target for integrating sensory and contextual information. For example, Mandairon et al. (2014) created a paradigm associating an odorant with a visual context. They found that exposing the animal to the context alone was able to produce odorant-seeking behavior and, at the OB level, activated a pattern of GCs activity similar to that induced by the odorant. On the other hand, by pharmacologically ablating inputs from other brain areas to the OB, they found that activation responses to the context in the OB depended on feedback inputs generated with higher-order areas (Mandairon et al., 2014). These experiments showed that upstream (sensory) and downstream (non-sensory) inputs contribute to cellular connectivity within the OB and to the higher-order inputs that GCs receive. Therefore, the newborn olfactory neurons integrate within a complex memory-supporting circuit. In the hippocampus, newborn neurons generate new synaptic contacts capable of disrupting previously established synapses (Toni et al., 2008). Then, it is likely that such disruptions affect previously established memory engrams; therefore, the hippocampus memory suffers a substantial degradation when neurogenesis rates increase (Akers et al., 2014; Epp et al., 2016). The newborn neurons of the OB integrate into circuits that process olfactory and contextual information; the addition of these new cells to the circuits likely generates a similar degradation effect as the one observed in the hippocampus. This continuous neuron addition should be able to disrupt fear-associated memories, as in the hippocampus (Akers et al., 2014) (Figure 2B).

Adams et al. (2019) developed a computational model that makes specific predictions for cortico-bulbar network connectivity that supports learning through exposure to odor and environmental contexts. This model predicts that the receptive fields of granule cells for sensory and cortical inputs are highly correlated. In this model, cortical cells can respond to a learned odor to perform disynaptic inhibitory control, specifically on the principal bulbar cells responding to that odor. For this, the reciprocal nature of granule cell synapses with principal cells is essential, and an increase in their number can generate an excess of inhibition to the reciprocal circuit that can decrease the fear responses of animals exposed to odors paired with aversive stimuli.

Based on the above, we support the idea that the engram maintenance in the OB, even amidst cell turnover, fundamentally relies on the reciprocal modulation between bulbar cells and higher-order structures. In these engrams, we should incorporate the ones linked to odor-associated fear responses. Although there are already works that support this idea, much remains to be done to describe the cognitive modulations generated by the addition and ablation of newborn neurons and the specific or multiple mechanisms, plastic, electrophysiological, or molecular, that explain the olfactory neurogenesis modulation of odor-associated fear learning.

## Author contributions

MS-B: Conceptualization, Writing – original draft, Writing – review & editing. GL-O: Writing – review & editing, Visualization. PD: Writing – review & editing. AM-C: Writing – review & editing, Conceptualization, Funding acquisition, Writing – original draft.

## Funding

The author(s) declare financial support was received for the research, authorship, and/or publication of this article. We appreciate the funding of DGAPA-UNAM, PAPIIT project IA205723 to AM-C. We appreciate the graduate scholarship to the National Council of Science and Technology (CONACyT) to GL-O.

## Conflict of interest

The authors declare that the research was conducted in the absence of any commercial or financial relationships that could be construed as a potential conflict of interest.

## Publisher’s note

All claims expressed in this article are solely those of the authors and do not necessarily represent those of their affiliated organizations, or those of the publisher, the editors and the reviewers. Any product that may be evaluated in this article, or claim that may be made by its manufacturer, is not guaranteed or endorsed by the publisher.
